# Treatments for blunt chest trauma and their impact on patient outcomes and health service delivery

**DOI:** 10.1186/s13049-015-0091-5

**Published:** 2015-02-08

**Authors:** Annalise Unsworth, Kate Curtis, Stephen Edward Asha

**Affiliations:** Trauma Department, St George Hospital, Gray Street, Kogarah, NSW 2217 Australia; Sydney Nursing School, University of Sydney, Sydney, NSW Australia; Faculty of Medicine, University of New South Wales, Kensington, NSW Australia; Department of Emergency, St George Hospital, Gray Street, Kogarah, NSW 2217 Australia

**Keywords:** Rib fractures, Management interventions, Patient and health outcomes

## Abstract

Blunt chest trauma is associated with a high risk of morbidity and mortality. Complications in blunt chest trauma develop secondary to rib fractures as a consequence of pain and inadequate ventilation. This literature review aimed to examine clinical interventions in rib fractures and their impact on patient and hospital outcomes. A systematic search strategy, using a structured clinical question and defined search terms, was performed in MEDLINE, EMBASE, CINAHL and the Cochrane Library. The search was limited to studies of adult humans from 1990-March 2014 and yielded 977 articles, which were screened against inclusion/exclusion criteria. A hand search was then performed of the articles that met the eligibility criteria, 40 articles were included in this review. Each article was assessed using a quantitative critiquing guideline. From these articles, interventions were categorised into four main groups: analgesia, surgical fixation, clinical protocols and other interventions. Surgical fixation was effective in patients with flail chest at improving patient outcomes. Epidural analgesia, compared to both patient controlled analgesia and intravenous narcotics in patients with three or more rib fractures improved both hospital and patient outcomes, including pain relief and pulmonary function. Clinical pathways improve outcomes in patients ≥ 65 with rib fractures. The majority of reviewed papers recommended a multi-disciplinary approach including allied health (chest physiotherapy and nutritionist input), nursing, medical (analgesic review) and surgical intervention (stabilisation of flail chest). However there was a paucity of evidence describing methods to implement and evaluate such multidisciplinary interventions. Isolated interventions can be effective in improving patient and health service outcomes for patients with blunt chest injuries, however the literature recommends implementing strategies such as clinical pathways to improve the care and outcomes of thesetre patients. The implementation of evidence-practice interventions in this area is scarce, and evaluation of interventions scarcer still.

**Electronic supplementary material:**

The online version of this article (doi:10.1186/s13049-015-0091-5) contains supplementary material, which is available to authorized users.

## Introduction

Blunt chest trauma is associated with a high risk of morbidity and mortality [1]. Rib fractures constitute a major part of blunt chest trauma and each additional rib fracture is associated with an increasing likelihood of developing complications [2,3]. Each additional rib fracture in the elderly population increases the odds of mortality by 19% and of developing pneumonia by 27% [3,4]. Respiratory complications develop with rib fractures as a consequence of splinting of the thorax from pain and mechanical instability resulting in inadequate ventilation [5]. Even an isolated rib fracture is associated with significant consequences, particularly in the older population [6,7]. This causes decreased lung volumes, atelectasis, and may progress to pneumonia, respiratory failure, need for prolonged ventilation and possible death [8]. Moreover reduced mobility in blunt chest trauma increases the likelihood of venous thrombosis [9,10].

Management of blunt chest trauma focuses on a combination of effective analgesia, surgical fixation, chest physiotherapy, respiratory care and early mobilisation [11,12]. If rib fracture pain in blunt chest trauma is not treated in a timely manner, complications may result in death, long-term pulmonary impairment, increased hospital length of stay (LOS) and increased use of health-care resources [1]. Inadequate or delayed pain relief is well known to cause people to eat less, sleep poorly, undergo psychological stress, experience restricted movement, and be unable to participate in normal activities including work [13]. Although literature reviews exist for some specific interventions such as surgical rib fixation and epidural analgesia, there are no published reviews evaluating all potential treatment options for blunt chest trauma. The purpose of this review is to examine the literature around interventions in blunt chest trauma to prevent complications and death.

## Review

### Research question

To review the treatments for blunt chest trauma and their impact on patient and hospital outcomes. Each term is defined, using the population, intervention, comparison, outcome (PICO) framework [14], in Table 1.

**Table 1:** **PICO research terms** [14]

**Population**	**Adult - blunt chest trauma**	**Sternal fractures, rib fractures, blunt chest injury, thoracic injury,**
Intervention	Multidisciplinary intervention	Models of care, management intervention, care practices, care protocols
Comparison	Other intervention	
Outcome	Patient and health outcomes	Mortality, pneumonia, pneumothorax, haemothorax, hospital length of stay, ICU stay, DVT, PE, costings, treatment outcome

ICU: Intensive care unit; DVT: deep venous thrombosis; PE: pulmonary embolism.

## Methods

An integrative review was conducted, which enabled the inclusion of a diverse range of study designs. The review process involved a search of the current literature, evaluation and categorisation of the data, and analysis of the groups [15]. A scoping search was conducted in the Medline Database to determine MESH terms appropriate to the research question. A search was conducted of the Cochrane, Medline, EMBASE and CINAHL databases in March 2014 using search terms including “thoracic injuries”, “rib fractures”, “mortality”, “pneumonia”, “outcome assessment” and “length of stay” [see Additional file 1: Tables S2 and S3] includes a full list of search terms and the databases searched. The initial search was performed without language restrictions or limitations to research design. The initial search was limited to studies of humans, adults and from 1990 onwards. This time-frame was chosen due to changes in interventions, including ventilation and surgical fixation based on a better understanding of blunt chest injury pathophysiology [16]. The initial search was limited to blunt chest trauma and did not focus on specific individual injury types. Patient outcomes included complications such as pneumonia, the duration of mechanical ventilation, pain level and mortality. Health service delivery referred to the access to interventions and the provision of multidisciplinary care, delivery outcomes included cost and hospital length of stay.

The search yielded 1107 articles, of which 130 were duplicates. The remaining 977 titles and abstracts were inspected to determine if they fulfilled our preliminary inclusion criteria: (1) original research, (2) blunt chest trauma, including articles that enrolled a mixture of blunt and penetrating chest trauma, or polytrauma, (3) any intervention for the treatment of blunt chest trauma, (4) included a comparator, (5) contained measured outcomes, and (6) the abstract was in English. 65 potentially relevant articles were identified. The full text articles were then obtained and reviewed by each author to ensure all inclusion and exclusion criteria were met. One article was unable to be retrieved, as a full manuscript had not been published.

**Figure 1:**
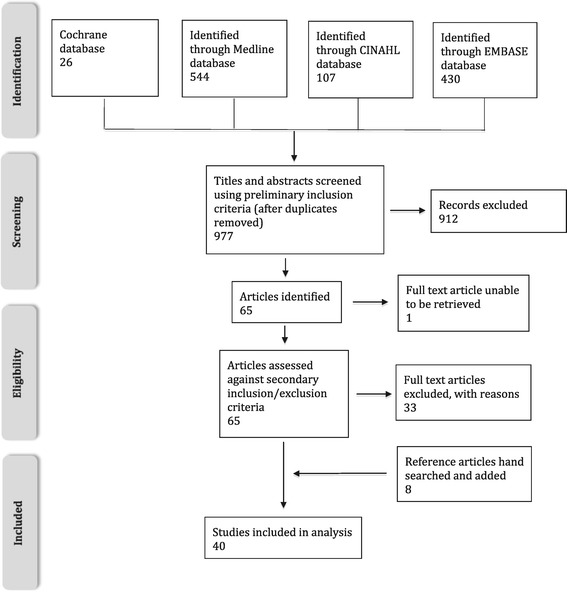
**Prisma diagram representing the search and screen process** [19]**.**

Articles were excluded if patient outcomes were primarily dependent on the speed and accessibility of advanced trauma surgical services, rather than on interventions aimed at preventing the complications of pain, immobility and respiratory impairment. For example articles evaluating patients with catastrophic injury requiring emergency thoracotomy were excluded due to the high rates of extrathoracic injuries, and the low baseline survival rates [17,18]. 32 articles remained after review of the full text for eligibility criteria. A hand search was conducted of the reference lists of these articles and eight further articles were included. The screening process is highlighted in Figure 1 [19]. The 40 remaining articles were analysed and are summarised [see Additional file 2: Table S4]. Each article was assessed using a quantitative critiquing guideline [20] to assess the quality of the study. Secondly, using the US Preventive Services Task Force guidelines, the level of evidence was rated as good, fair or poor [21]. Evidence is rated based on parameters for evaluating the internal validity of the different study designs. A good study meets all criteria for that study design, a fair study does not meet all criteria but it has no fatal flaw that invalidates its results, and a poor study contains a fatal flaw [21]. Information extracted included level of evidence, patient group, outcomes, findings and limitations.

## Results

The 40 reviewed articles originated from 12 different countries with varying health systems, including USA, Germany, Australia, Italy and Egypt. Research methods ranged from case series to randomised controlled trials. Using the critiquing guidelines for level of evidence, 7/40 articles were assessed as poor, 20/40 as fair and 13/40 as good.

The treatments for blunt chest trauma identified were grouped into four main categories: surgical stabilisation of rib fractures, analgesia for effective pain relief, the implementation of clinical protocols and multidisciplinary interventions, and other interventions, including ventilation and video assisted thoracoscopy surgery. The main outcome measures reported were ventilator dependent days, cost, complications including pneumonia, mortality, pain scores and hospital and intensive care unit length of stay (ICU-LOS). Surgical fixation was limited to patients with flail chest and the majority of analgesic modalities were in patients with three or more rib fractures following blunt chest injury. Multidisciplinary interventions (clinical pathways) were evaluated in patients 45 years and older with four or more rib fractures or patients 65 years and older with three or more rib fractures.

### Surgical stabilisation

Flail chest, where at least three consecutive ribs are fractured in two or more places, compromises thoracic cage integrity and is associated with significant morbidity and mortality [22]. This is particularly true in the elderly, those with bilateral flail and patients with pulmonary contusion as it is associated with a higher rate of complications and a longer period of mechanical ventilation [23-26]. Surgical rib fixation (SRF) is a treatment for flail chest as it provides chest wall stability. Retrospective studies have noted a significant decrease in mechanical ventilation requirements after surgical fixation [22,25,27]. Through decreasing the time required on mechanical ventilation, surgical fixation prevents ventilator-acquired pneumonia [25,28], a common complication of prolonged intubation [24,25].

Three randomised trials provided the best evidence for the benefits of surgical rib fracture fixation. Surgical fixation was identified as a treatment for patients with flail chest requiring mechanical ventilation and is associated with a decrease in ICU-LOS, fewer days of mechanical ventilation and cost savings compared to non-operative management [28-30]. Tanaka et al. [28] randomised 37 consecutive flail chest patients, intubated in emergency and requiring ventilation, to either surgical stabilisation or conservative internal pneumatic stabilisation. Internal pneumatic stabilisation involves providing positive end-expiratory pressure ventilation through intubation and mechanical ventilation or non-invasive ventilation devices. The surgical rib fixation group demonstrated decreased days of ventilator dependence, and shorter ICU-LOS [28]. There was a lower incidence of pneumonia, a higher return to full time work at six months, and less persistent pain at six and 12 months in those receiving surgery [28]. Granetzny et al. [29] and Marasco et al. [30] also found that patients who received surgical treatment had significantly fewer days of mechanical ventilation and a shorter hospital and ICU-LOS. The estimated cost savings ranged from USD 10,000 [28] to AUD 14,443 [30] per patient with surgical rib fixation as a result of the decrease in ICU-LOS [30]. None of the studies were large enough to draw conclusions on the effect of this intervention on thromboembolism and death.

### Analgesia

Pain in blunt chest trauma is associated with restricted ventilatory function, which can lead to serious complications [5]. Multiple methods of analgesia have been evaluated and compared in the rib fracture population, including non-steroid anti-inflammatory medications, epidural catheters, intravenous narcotics, patient controlled analgesia (PCA), lidocaine patches, intercostal blocks and paravertebral blocks [8,12,31-36].

The most predominate mode of analgesia reported was epidural catheters. The available evidence for patients with three or more rib fractures suggests that epidural analgesia provides more effective pain relief in comparison with other analgesic modalities, and it is most applicable to patients with functional respiratory compromise secondary to pain [3]. There was less evidence for patients with one or more fractured ribs, and studies were limited to > 65 years, however, retrospective studies suggest that patients given epidural analgesia compared to IV narcotics reduces mortality [37].

Comparing epidural analgesia with intravenously administered opioids or patient controlled analgesia, Mackersie et al. [32] and Bulger et al. [38] in randomised controlled trials respectively reported that epidural analgesia was associated with a mild improvement in pulmonary mechanics, and reduced rates of pneumonia and the duration of mechanical ventilation. In two retrospective cohort studies, Gage et al. [11] with 836 patients and Wisner et al. [37] with 307 patients, showed that epidural analgesia was associated with a reduction in short and long term mortality compared to those using other analgesic modalities. Compared to PCA, epidural analgesia is associated with lower pain scores [36,39], however, as PCA pain control is reliant upon a degree of patient understanding in how to deliver their analgesia, higher pain scores in the PCA group may indicate the importance of patient selection when choosing PCA [36,39]. In a trauma patient with fractures, epidurals can be technically difficult to place, and are contraindicated in patients with coagulopathy [31]. Additionally, epidural catheters are associated with a number of complications including headache, respiratory depression, infection, neurological injury and epidural haematoma [40].

Two other analgesic techniques include lidocaine patches and intercostal and paravertebral blocks. Lidocaine patches involve placing an adhesive patch containing 5% lidocaine directly over the site of injury/pain [41]. When evaluated in a randomised double-blind, placebo controlled trial of 58 patients, no improvement in pain control or hospital length of stay could be demonstrated [35]. Whilst lidocaine patches are easy to use and are fairly benign, they may offer little or no pain control, and further research is necessary before they can be recommended as an adjunct for pain control in this setting. Paravertebral and intercostal nerve blocks improved pain scores at rest and on coughing compared to the initial treatment [31,34,42]. However, neither nerve block was evaluated directly with an alternate analgesic technique such as epidurals or PCA.

Larger randomised controlled trials comparing epidural analgesia with intravenous narcotics, patient controlled analgesia and nerve blocks are required to draw a firm conclusion on the overall effectiveness of epidural analgesia. Current limitations exist, particularly due to the small sample size in a number of studies [32,38], meaning that the rare but severe complications are not examined.

### Clinical pathways

Rib fracture clinical pathways were first documented by Sesperez et al. in 2001 [43], and aim to streamline patient care and provide guidelines for the different interventions required in rib fracture treatment. The four clinical pathways that have been evaluated in the literature, show an improvement in patient and hospital outcomes post protocol introduction, by reducing patient complications and hospital costs [43-47]. These pathways have been shown to be applicable in patients with ≥1 fractured rib [43] and improve outcomes in patients ≥ 65 with one or more rib fractures [46].

A multidisciplinary pathway improved outcomes for patients and decreased ICU and Hospital LOS, pneumonia and mortality [47]. This pathway applied to patients greater than 45 with four or more rib fractures and involved the pain service for analgesia review, respiratory team for medical care, physiotherapy for deep breathing exercises, nutritionist to monitor the nutritional status of patients and nurse practitioner to ensure continuity of care. Patients on this pathway were given significantly more aggressive pain management, including prescription of PCA and epidural catheters, which could account for the improvement in outcomes [47]. Two other clinical protocols demonstrated a decrease in hospital LOS [46] and emergency department re-attendance in the post-implementation period of this protocol [45].

Adrales et al. [48] examined a practice guideline for thoracostomy tubes, showing that post-implementation of the practice guideline, there were less chest radiographs and fewer days of thoracostomy tubes. However, contrary to the guidelines, 26% of patients received more than the recommended 24 hours of antibiotic therapy and 45% had chest radiographs before thoracostomy tube removal. This suggests the difficulties in implementing and complying with clinical guidelines.

### Other interventions

The treatment of rib fractures includes primary modalities such as surgical rib fixation, analgesia and ventilation, as well as secondary treatments for complications such as thorocostomy tubes and video-assisted thoracoscopic surgery.

Ventilation is an important treatment modality for rib fracture management in order to mitigate shallow breathing associated with rib fractures [49,50]. Bolliger et al. [49] and Walz et al. [50] compared CPAP to endotracheal intubation and mechanical ventilation, demonstrating that patients treated with CPAP had a shorter stay in ICU and hospital, and developed significantly fewer complications. Incentive spirometry, to promote deep breathing, was shown by Shukla et al. to reduce morbidity [42]. In order to be effective, both CPAP and spirometry require adequate analgesia [51].

For the treatment of a persistent pneumothorax or haemothorax after blunt chest injury, video-assisted thoracoscopy surgery (VATS) has been developed [52]. Fabbrucci et al. [52] compared patients with only a chest tube to patients with a chest tube and VATS. Their results were inconclusive, for whilst both groups had similar recovery time, the two groups had different levels of injury severity. Smith et al. [53] compared patients who received VATS ≤ 5 days after injury and > 5 days after injury. Patients treated ≤ 5 days after injury had a lower rate of conversion to an open procedure and a lower overall hospital LOS. Both these studies suggest that VATS is an effective procedure for treating complications of blunt thoracic injury [52,53].

## Discussion

**Figure 2:**
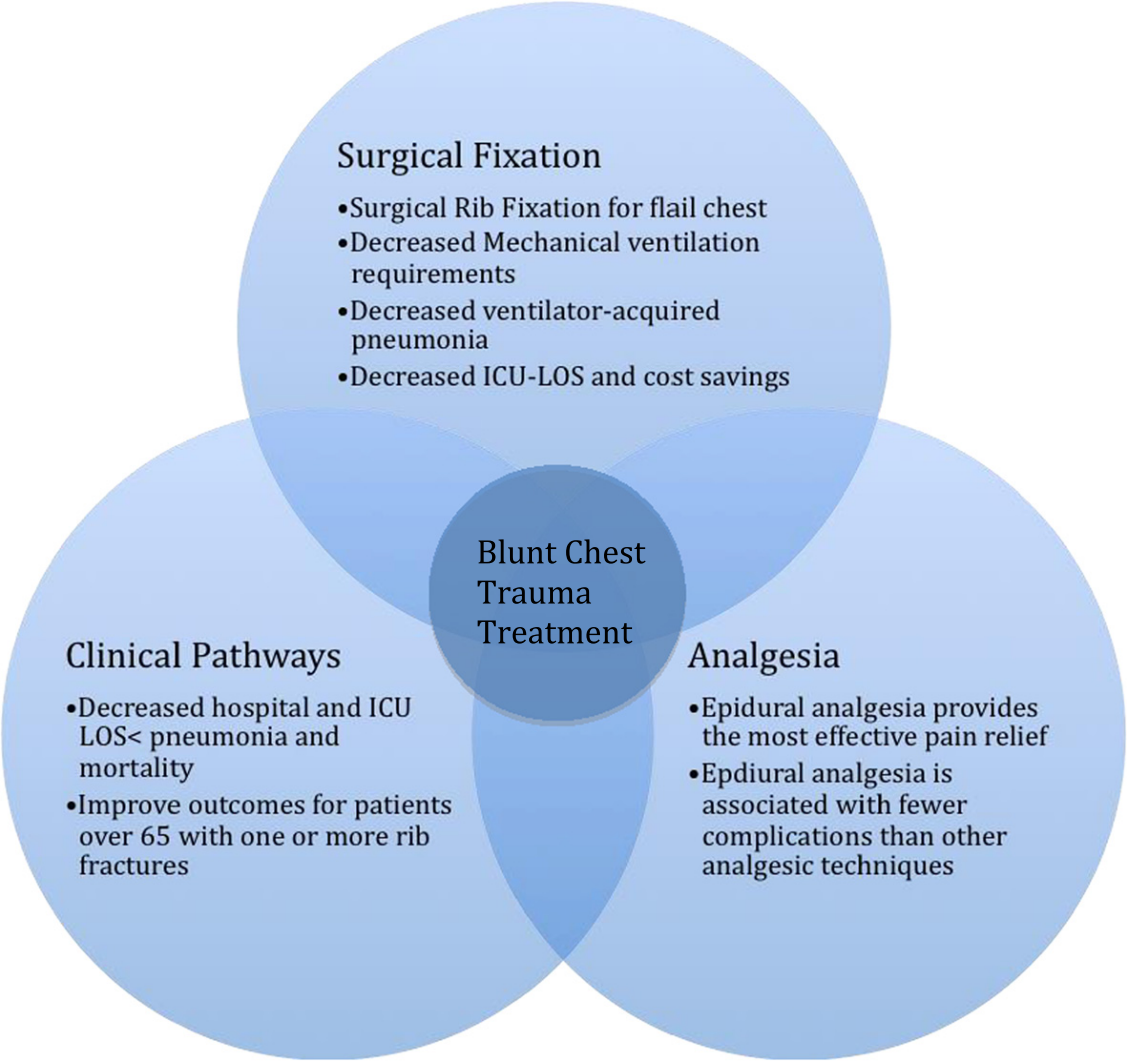
**Key findings in blunt chest trauma treatment.** ICU: Intensive care unit; LOS: Length of stay.

Blunt thoracic trauma has a significant impact on morbidity and mortality when left untreated [1]. Many different treatment modalities are used to reduce the complications associated with rib fractures, however, their efficacy is often unclear. This literature review provides a comprehensive examination of the different treatment modalities involved and their individual and relative effectiveness. The key findings of this review are highlighted in Figure 2. The results show that analgesia, particularly in the form of epidurals, surgical fixation and ventilation are single modal interventions effective at improving outcomes. However, difficulties exist in ensuring that patients receive a full spectrum of care, hence clinical pathways have been developed in order to organise this care for the patient. The findings of this review are limited by the quality of evidence. Only 10 of the 40 articles were prospective randomised trials. Most trials were limited by small sample size, and by selection bias in retrospective studies. A summary of each treatment option and the strength of the surrounding literature is illustrated [see Additional file 3: Table S5].

### Evolution of interventions

The literature describing rib fracture treatment has developed from the delivery of single modal interventions to broader multidisciplinary care, encompassing multiple interventions. In the past, rib belt treatments were popular for the treatment of rib fractures [54]. However, their use has been superseded by improvements in ventilation and surgical fixation [31]. Due to the development of positive pressure ventilation in the 1950s, mechanical ventilation became a mainstay in the treatment of rib fractures [55]. Due to the increasing evidence of the association of intubation and mechanical ventilation with complications including pneumonia and death [56], less invasive forms of respiratory support have been developed [57,58]. Despite association with increased complications, mechanical ventilation is applicable for patients with flail chest and severe concomitant injuries or patients with persistent respiratory compromise or failure after sufficient analgesia [59]. Surgical fixation has had a recent increase in interest particularly in the treatment of flail chest, where it seems to improve outcomes [28-30]. However, its broader application is limited by the difficulties in defining appropriate population groups for treatment and the current low incidence of rib fixation in trauma centres [22,60].

Chest physiotherapy, including incentive spirometry, and CPAP have decreased complications in rib fracture treatment [31,50,61]. This literature review returned limited evidence into the role of chest physiotherapy and allied health in the outcomes of patients with blunt chest trauma and primarily focused on patients with multiple rib fractures. Rib fractures are reported to be the most common clinical fracture in older people [62], and this demographic is the most at risk of rib-fracture-related morbidity [7,63-65]. Rapid mobilisation through physiotherapy is considered a key factor in preventing complications, including pneumonia, respiratory failure and ARDS [65]. However to facilitate these interventions, effective pain control is necessary to allow for deep breathing chest physiotherapy and improved lung function [66]. Further evidence is required in determining the effectiveness of combining therapies, including mobilisation, respiratory care and pain management [5,61]. Additionally, patient outcomes are impacted by other treatment interventions, particularly in the polytrauma patient, such as fluid resuscitation [67]. Consideration needs to be made by multidisciplinary teams for the impact of other therapies on blunt chest trauma.

The literature recommends implementing clinical pathways to coordinate trans-disciplinary interventions, improve compliance with recommended treatment and ensure consistency in the delivery of interventions [43]. Successful clinical pathways standardise practice, translate research into practice and improve inter-professional co-ordination [68]; and they are known to improve patient and health service outcomes across a number of other clinical conditions, such as planned knee replacement surgery [69], stroke and heart failure [70]. An important limitation of clinical pathways can be an inflexibility for tailoring care to individual patient’s needs [71]. For example, eligibility for a pathway require a certain number of ribs to be demonstrably fractured, but we know more than 50% of rib fractures cannot be seen on x-ray [72]. Particularly amongst the elderly, the physiologic consequences of clinically apparent rib fractures may be very similar whether or not a fracture is radiologically visible, and so should be considered for inclusions in these pathways. The evaluations have been limited to the effectiveness of the pathway as a whole, rather than the clinical interventions within the pathway that may contribute to improved outcomes. Todd et al. [47] suggested that the aggressive use of the pain service was integral to the benefits elicited in the post-pathway group, while Sahr et al. [46] attributed the benefits to the ability of the trauma team to coordinate multidisciplinary care. Further research is required to determine which aspects of the clinical pathways are effective at improving patient outcomes.

### Implementing interventions and organising patient care

To ensure that clinical pathways are effectively implemented, continual reinforcement and education are required to ensure the pathways are activated [43]. There are many barriers to the implementation of multidisciplinary care pathways including reluctance to change and lack of suitable existing evidence for their implementation [73]. As a variety of interventions are effective in treating rib fractures involving multiple medical and allied health disciplines, to ensure coordination, implementation, monitoring and evaluation of recommended care, an organised system is required. One such model is the trauma nurse case manager, which is known to be effective at reducing complications and improving the time to allied health interventions [74]. Trauma case managers are integral to facilitate the achievement of outcomes and reinforcing the implementation of clinical pathways [43]. Other forms of organising patient care include multidisciplinary rounds, whereby medical, surgical and allied health professionals meet to discuss patient care. These have also been shown to decrease hospital length of stay and streamline the care of trauma patients [75].

This review has highlighted the need for the delivery of multidisiciplinary interventions such as a “care bundle”. “Care bundles” are a uniform set of evidence based practices specific to a clinical presentation theater considered by the attending team [76]. A “care bundle” for blunt chest trauma could be comprised of oxygen therapy, physiotherapy, analgesia and trauma care coordination. In determining patients eligible for a “care bundle” consideration needs to be made of the presence of lung injury, concomitant injuries, age, number of rib fractures and underlying respiratory disease [77,78].

### Sources of further literature

Similar pulmonary complications exist between rib fractures in cardiothoracic surgery and rib fractures following blunt chest trauma [79]. Lawrence et al. [79] in a systematic review analysed interventions to prevent postoperative complications after non-cardiothoracic surgery. A synthesis of 16 studies concluded that lung expansion interventions (for example, incentive spirometry, deep breathing exercises and continuous positive airway pressure) reduce pulmonary postoperative complications. These results are similar to those in rib fracture interventions [50,62]. Cohn et al. [80] supports the role of clinical pathways, demonstrating that for all uncomplicated cardiac surgery patients, a clinical pathway reduces costs and hospital LOS in patients undergoing cardiac surgery. Review and adaptation of interventions after cardiothoracic surgery may add to the knowledge base of treatments in blunt chest trauma.

### Limitations

There was no literature included in this review on physiotherapy and high flow nasal prongs, despite both treatments being a mainstay in rib fracture management [81,82]. Other ventilation treatments including CPAP had limited evidence, and a comparison and definition of the appropriate CPAP and HFNP patient is required. Further randomised controlled studies are required into patient controlled analgesia and other analgesic techniques such as nerve blocks. Additionally, further research is needed into clinical pathways that take into account a greater number of risk factors [63,64].

## Conclusion

A broad amount of literature exists surrounding interventions in blunt chest trauma. The three treatment modalities that have significant evidence for their benefits are surgical rib fixation, epidural analgesia and trans-disciplinary clinical pathways. Collectively, these improve hospital outcomes including ICU and hospital LOS as well as patient outcomes including mortality and morbidity in patients with blunt chest trauma. Systems that address each component of multidisciplinary care and ensure their implementation would benefit patient and hospital outcomes. Further evidence is required in determining the effectiveness of combining therapies, including mobilisation, respiratory care and pain management.

## Additional files

Additional file 1: Table S2. Search Strategy used in database search. **Table S3.** Summary of literature searched. Additional file 2: Table S4. Overview of the Studies. Additional file 3: Table S5. Summary of each treatment option and strength of the literature [83-88].

## Abbreviations

ICU: Intensive care unit
LOS: Length of stay
IV: Intravenous
ISS: Injury severity score
SRF: Surgical rib fixation
PCA: Patient controlled analgesia
PE: Pulmonary embolism
CPAP: Continuous positive airway pressure
VATS: Video assisted thoracoscopic surgery

## References

[1] Bulger EM, Arneson MA, Mock CN, Jurkovich GJ (2000). Rib fractures in the elderly. J Trauma-Injury Infec Crit Care.

[2] Lee RB, Bass SM, Morris JA, MacKenzie EJ (1990). Three or more rib fractures as an indicator for transfer to a Level I trauma center: a population-based study. J Trauma-Injury Infect Crit Care.

[3] Yeh DD, Kutcher ME, Knudson MM, Tang JF (2012). Epidural analgesia for blunt thoracic injury–which patients benefit most?. Injury.

[4] Wardhan R (2013). Assessment and management of rib fracture pain in geriatric population: an ode to old age. Curr Opin Anaesthesiol.

[5] Easter A (2001). Management of patients with multiple rib fractures. Am J Crit Care.

[6] Barnea Y, Kashtan H, Skornick Y, Werbin N (2002). Isolated rib fractures in elderly patients: mortality and morbidity. Can J Surg.

[7] Elmistekawy E, Hammad AA (2007). Isolated rib fractures in geriatric patients. Annals of Thoracic Med.

[8] Bayouth L, Safcsak K, Cheatham ML, Smith CP, Birrer KL, Promes JT (2013). Early intravenous ibuprofen decreases narcotic requirement and length of stay after traumatic rib fracture. Am Surg.

[9] Geerts WH, Code KI, Jay RM, Chen E, Szalai JP (1994). A prospective study of venous thromboembolism after major trauma. N Engl J Med.

[10] Brathwaite C, Mure A, O’Malley K, Spence R, Ross S (1993). Complications of anticoagulation for pulmonary embolism in low risk trauma patients. CHEST J.

[11] Gage A, Rivara F, Wang J, Jurkovich GJ, Arbabi S (2014). The effect of epidural placement in patients after blunt thoracic trauma. J Trauma Acute Care Surg.

[12] Mohta M, Verma P, Saxena AK, Sethi AK, Tyagi A, Girotra G (2009). Prospective, randomized comparison of continuous thoracic epidural and thoracic paravertebral infusion in patients with unilateral multiple fractured ribs–a pilot study. J Trauma-Injury Infect Crit Care.

[13] Møiniche S, Kehlet H, Dahl JB (2002). A qualitative and quantitative systematic review of preemptive analgesia for postoperative pain relief: the role of timing of analgesia. Anesthesiology.

[14] Sackett D, Richardson W, Rosenberg W, Haynes R (1997). Evidence-based medicine: how to practice and teach EBM.

[15] Whittemore R, Knafl K (2005). The integrative review: updated methodology. J Adv Nurs.

[16] Karmakar MK, Ho AM-H (2003). Acute pain management of patients with multiple fractured ribs. J Trauma-Injury, Infect Crit Care..

[17] Balkan ME, Oktar GL, Kayi-Cangir A, Ergul EG (2002). Emergency thoracotomy for blunt thoracic trauma. Annals Thoracic Cardiovasc Surg.

[18] Kalina M, Teeple E, Fulda G (2009). Are there still selected applications for resuscitative thoracotomy in the emergency department after blunt trauma?. Del Med J.

[19] Moher D, Liberati A, Tetzlaff J, Altman DG (2009). Preferred reporting items for systematic reviews and meta-analyses: the PRISMA statement. Ann Intern Med.

[20] Polit-O’Hara D, Hungler BP (1993). Essentials of nursing research: methods, appraisal, and utilization.

[21] Harris RP, Helfand M, Woolf SH, Lohr KN, Mulrow CD, Teutsch SM (2001). Current methods of the US preventive services task force: a review of the process. Am J Prev Med.

[22] Doben AR, Eriksson EA, Denlinger CE, Leon SM, Couillard DJ, Fakhry SM (2014). Surgical rib fixation for flail chest deformity improves liberation from mechanical ventilation. J Crit Care.

[23] Borman JB, Aharonson-Daniel L, Savitsky B, Peleg K (2006). Unilateral flail chest is seldom a lethal injury. Emerg Med J.

[24] Freedland M, Wilson RF, Bender JS, Levison MA (1990). The management of flail chest injury: factors affecting outcome. J Trauma-Injury Infect Crit Care.

[25] Althausen PL, Shannon S, Watts C, Thomas K, Bain MA, Coll D (2011). Early surgical stabilization of flail chest with locked plate fixation. J Orthop Trauma.

[26] Voggenreiter G, Neudeck F, Aufmkolk M, Obertacke U, Schmit-Neuerburg KP (1996). Outcome of operative chest wall stabilization in flail chest with or without pulmonary contusion. [German]. Unfallchirurg.

[27] Nirula R, Allen B, Layman R, Falimirski ME, Somberg LB (2006). Rib fracture stabilization in patients sustaining blunt chest injury. Am Surg.

[28] Tanaka HYT, Yamaguti Y, Shimizu S, Goto H, Matsuda H, Shimazaki S (2002). Surgical stabilization of internal pneumatic stabilization? A prospective randomized study of management of severe flail chest patients. J Trauma.

[29] Granetzny A, El-Aal MA, Emam E, Shalaby A, Boseila A (2005). Surgical versus conservative treatment of flail chest. Eval Pulm Status Interact Cardiovas Thoracic Surg.

[30] Marasco SF, Davies AR, Cooper J, Varma D, Bennett V, Nevill R (2013). Prospective randomized controlled trial of operative rib fixation in traumatic flail chest. J Am Coll Surg.

[31] Truitt MS, Murry J, Amos J, Lorenzo M, Mangram A, Dunn E (2011). Continuous intercostal nerve blockade for rib fractures: Ready for primetime?. J Trauma - Injury Infect Crit Care.

[32] Mackersie RC, Karagianes TG, Hoyt DB, Davis JW (1991). Prospective evaluation of epidural and intravenous administration of fentanyl for pain control and restoration of ventilatory function following multiple rib fractures. J Trauma-Injury, Infect Crit Care..

[33] Asha SE, Curtis KA, Taylor C, Kwok A (2013). Patient-controlled analgesia compared with interval analgesic dosing for reducing complications in blunt thoracic trauma: a retrospective cohort study. Emerg Med J.

[34] Karmakar MK, Critchley LA, Ho AM-H, Gin T, Lee TW, Yim AP (2003). Continuous thoracic paravertebral infusion of bupivacaine for pain management in patients with multiple fractured ribs. CHEST J.

[35] Ingalls NK, Horton ZA, Bettendorf M, Frye I, Rodriguez C (2010). Randomized, double-blind, placebo-controlled trial using lidocaine patch 5% in traumatic rib fractures. J Am Coll Surg.

[36] Moon MR, Luchette FA, Gibson SW, Crews J, Sudarshan G, Hurst JM (1999). Prospective, randomized comparison of epidural versus parenteral opioid analgesia in thoracic trauma. Ann Surg.

[37] Wisner DH (1990). A stepwise logistic regression analysis of factors affecting morbidity and mortality after thoracic trauma: effect of epidural analgesia. J Trauma-Injury Infect Crit Care.

[38] Bulger EM, Edwards T, Klotz P, Jurkovich GJ (2004). Epidural analgesia improves outcome after multiple rib fractures. Surgery.

[39] Wu CL, Jani ND, Perkins FM, Barquist E (1999). Thoracic epidural analgesia versus intravenous patient-controlled analgesia for the treatment of rib fracture pain after motor vehicle crash. J Trauma-Injury Infect Crit Care.

[40] Auroy Y, Narchi P, Messiah A, Litt L, Rouvier B, Samii K (1997). Serious complications related to regional anesthesia: results of a prospective survey in France. Anesthesiology.

[41] Zink KA, Mayberry JC, Peck EG, Schreiber MA (2011). Lidocaine patches reduce pain in trauma patients with rib fractures. Am Surg.

[42] Shukla AN, Ghaffar ZBA, Auang AC, Rajah U, Tan L (2008). Continuous paravertebral block for pain relief in unilateral multiple rib fracture: a case series. Acute Pain.

[43] Sesperez J, Wilson S, Jalaludin B, Seger M, Sugrue M (2001). Trauma case management and clinical pathways: prospective evaluation of their effect on selected patient outcomes in five key trauma conditions. J Trauma-Injury Infect Crit Care.

[44] Wilson S, Bin J, Sesperez J, Seger M, Sugrue M (2001). Clinical pathways–can they be used in trauma care. An Anal Patient Inj.

[45] Menditto VG, Gabrielli B, Marcosignori M, Screpante F, Pupita G, Polonara S (2012). A management of blunt thoracic trauma in an emergency department observation unit: pre-post observational study. J Trauma Acute Care Surg.

[46] Sahr SM, Webb ML, Renner CH, Sokol RK, Swegle JR (2013). Implementation of a rib fracture triage protocol in elderly trauma patients. J Trauma Nurs.

[47] Todd SR, McNally MM, Holcomb JB, Kozar RA, Kao LS, Gonzalez EA (2006). A multidisciplinary clinical pathway decreases rib fracture-associated infectious morbidity and mortality in high-risk trauma patients. Am J Surg.

[48] Adrales G, Huynh T, Broering B, Sing RF, Miles W, Thomason MH (2002). A thoracostomy tube guideline improves management efficiency in trauma patients. J Trauma-Inj Infect Crit Care.

[49] Bolliger CT, Van Eeden SF (1990). Treatment of multiple rib fractures. Randomized controlled trial comparing ventilatory with nonventilatory management. Chest..

[50] Walz M, Mollenhoff G, Muhr G (1998). CPAP-augmented spontaneous respiration in thoracic trauma. An alternative to intubation. Unfallchirurg..

[51] Gunduz M, Unlugenc H, Ozalevli M, Inanoglu K, Akman H (2005). A comparative study of continuous positive airway pressure (CPAP) and intermittent positive pressure ventilation (IPPV) in patients with flail chest. Emerg Med J.

[52] Fabbrucci P, Nocentini L, Secci S, Manzoli D, Bruscino A, Fedi M (2008). Video-assisted thoracoscopy in the early diagnosis and management of post-traumatic pneumothorax and hemothorax. Surg Endosc.

[53] Smith JW, Franklin GA, Harbrecht BG, Richardson JD (2011). Early VATS for blunt chest trauma: a management technique underutilized by acute care surgeons. J Trauma-Inj Infect Crit Care.

[54] Quick G (1990). A randomized clinical trial of rib belts for simple fractures. Am J Emerg Med.

[55] Avery E, Benson D, Morch E (1956). Critically crushed chests; a new method of treatment with continuous mechanical hyperventilation to produce alkalotic apnea and internal pneumatic stabilization. J Thorac Surg.

[56] Chiumello D, Coppola S, Froio S, Gregoretti C, Consonni D (2013). Noninvasive ventilation in chest trauma: systematic review and meta-analysis. Intensive Care Med.

[57] Shackford SR, Smith DE, Zarins CK, Rice CL, Virgilio RW (1976). The management of flail chest: a comparison of ventilatory and nonventilatory treatment. Am J Surg.

[58] Trinkle JK, Richardson JD, Franz JL, Grover FL, Arom KV, Holmstrom FM (1975). Management of flail chest without mechanical ventilation. Ann Thorac Surg.

[59] Pettiford BL, Luketich JD, Landreneau RJ (2007). The management of flail chest. Thorac Surg Clin.

[60] De Moya M, Bramos T, Agarwal S, Fikry K, Janjua S, King DR (2011). Pain as an indication for rib fixation: a bi-institutional pilot study. J Trauma - Inj, Infect Crit Care..

[61] Schwed AC, Sonnad SS, Holena DN, Pascual JL, Reilly PM, Sims CA (2013). Take a deep breath!: managing rib fractures in elderly trauma patients. J Surg Res..

[62] Barrett-Connor E, Nielson CM, Orwoll E, Bauer DC, Cauley JA. Epidemiology of rib fractures in older men: osteoporotic fractures in men (MrOS) prospective cohort study. BMJ. 2010;00:00 2010:340. 10.1136/bmj.c1069 PMC2839084 20231246

[63] Bergeron E, Lavoie A, Clas D, Moore L, Ratte S, Tetreault S (2003). Elderly trauma patients with rib fractures are at greater risk of death and pneumonia. J Trauma - Inj Infect Crit Care.

[64] Ziegler DW, Agarwal NN (1994). The morbidity and mortality of rib fractures. J Trauma.

[65] Sharma OP, Oswanski MF, Jolly S, Lauer SK, Dressel R, Stombaugh HA (2008). Perils of rib fractures. Am Surg.

[66] Winters BA. older adults with traumatic rib fractures: an evidence based approach to their care. Int J Trauma Nurs. 2009;16(2):93–7. 10.1097/JTN.0b013e3181ac9201 19543018

[67] Dutton RP, Mackenzie CF, Scalea TM (2002). Hypotensive resuscitation during active hemorrhage: impact on in-hospital mortality. J Trauma-Inj Infect Crit Care.

[68] Hunter B, Segrott J (2008). Re-mapping client journeys and professional identities: a review of the literature on clinical pathways. Int J Nurs Stud.

[69] Rotter T, Kugler J, Koch R, Gothe H, Twork S, van Oostrum JM (2008). A systematic review and meta-analysis of the effects of clinical pathways on length of stay, hospital costs and patient outcomes. BMC Health Serv Res.

[70] Vanhaecht K, Panella M, Van Zelm R, Sermeus W (2010). An overview on the history and concept of care pathways as complex interventions. Intl J Care Pathw.

[71] Goldberger JJ, Buxton AE (2013). Personalized medicine vs guideline-based medicine. Jama.

[72] Bansidhar BJ, Lagares-Garcia JA, Miller S (2002). Clinical rib fractures: are follow-up chest X-rays a waste of resources?. Am Surg.

[73] Campbell H, Hotchkiss R, Bradshaw N, Porteous M (1998). Integrated care pathways. BMJ: British Med J.

[74] Curtis K, Zou Y, Morris R, Black D (2006). Trauma case management: improving patient outcomes. Injury.

[75] Dutton RP, Cooper C, Jones A, Leone S, Kramer ME, Scalea TM (2003). Daily multidisciplinary rounds shorten length of stay for trauma patients. J Trauma-Inj Infect Crit Care.

[76] Resar R, Pronovost P, Haraden C, Simmonds T, Rainey T, Nolan T (2005). Using a bundle approach to improve ventilator care processes and reduce ventilator-associated pneumonia. Jt Comm J Qual Patient Saf.

[77] Huber S, Biberthaler P, Delhey P, Trentzsch H, Winter H, van Griensven M (2014). Predictors of poor outcomes after significant chest trauma in multiply injured patients: a retrospective analysis from the German Trauma Registry (Trauma Register DGU®). Scand J Trauma Resusc Emerg Med.

[78] Geiger EV, Lustenberger T, Wutzler S, Lefering R, Lehnert M, Walcher F (2013). Predictors of pulmonary failure following severe trauma: a trauma registry-based analysis. Scand J Trauma Resusc Emerg Med.

[79] Lawrence VA, Cornell JE, Smetana GW (2006). Strategies to reduce postoperative pulmonary complications after noncardiothoracic surgery: systematic review for the American College of Physicians. Ann Intern Med.

[80] Cohn MDLH, Rosborough RND, Fernandez MPAJ (1997). Reducing costs and length of stay and improving efficiency and quality of care in cardiac surgery. Ann Thorac Surg.

[81] Liman ST, Kuzucu A, Tastepe AI, Ulasan GN, Topcu S (2003). Chest injury due to blunt trauma. Eur J Cardiothorac Surg.

[82] Clini E, Ambrosino N (2005). Early physiotherapy in the respiratory intensive care unit. Respir Med.

[83] Ahmed Z, Mohyuddin Z (1995). Management of flail chest injury: internal fixation versus endotracheal intubation and ventilation. J Thorac Cardiovasc Surg.

[84] Hakim SM, Latif FS, Anis SG (2012). Comparison between lumbar and thoracic epidural morphine for severe isolated blunt chest wall trauma: a randomized open-label trial. J Anesth.

[85] Topcu I, Ekici Z, Sakarya M. [Comparison of clinical effectiveness of thoracic epidural and intravenous patient-controlled analgesia for the treatment of rib fractures pain in intensive care unit]. Ulusal Travma ve Acil Cerrahi Dergisi = Turkish J Trauma & Emerg Surg: TJTES. 2007;13(3):205–10. 17978895

[86] Kieninger AN, Bair HA, Bendick PJ, Howells GA (2005). Epidural versus intravenous pain control in elderly patients with rib fractures. Am J Surg.

[87] Pierre EJ, Martin P, Frohock J, Varon AJ, Barquist E (2005). Lumbar epidural morphine versus. Patient-controlled analgesia morphine in patients with multiple rib fractures. Anesthesiology.

[88] Lohr KN (2004). Rating the strength of scientific evidence: relevance for quality improvement programs. Int J Qual Health Care.

