# Supplementary material for: Treatments for blunt chest trauma and their impact on patient outcomes and health service delivery
Source: Scand J Trauma Resusc Emerg Med. 2015 Feb 8;23:17. doi: 10.1186/s13049-015-0091-5 (PMC4322452; doi:10.1186/s13049-015-0091-5)
Supplement: Additional file 1: Table S2. — Search Strategy used in database search. Table S3. Summary of literature searched. [file 13049_2015_91_MOESM1_ESM.docx]

**Additional File 1**

**Table S2: Search Strategy used in database search**

| Cochrane Database of Systematic Reviews/ Database of Abstracts of Reviews of Effects/ Cochrane central register of controlled trials | | |
| --- | --- | --- |
| 1 | Exp chest injury/ | 154 |
| 2 | Chest injury.tw | 17 |
| 3 | Thoracic injury.tw | 14 |
| 4 | Chest trauma.mp. [mp=ti, ot, tx, hw, kw ,ab, sh, ct] | 59 |
| 5 | Or/1-4 | 207 |
| 6 | Exp patient outcome | 4206 |
| 7 | Exp pneumonia/ | 2230 |
| 8 | Exp pneumothorax/ | 196 |
| 9 | Or/6-8 | 6560 |
| 10 | 5 and 9 | 26 |
| Medline Database | | |
| 1 | Thoracic injuries/ or flail chest/ or lung injury/ or rib fractures/ | 16334 |
| 2 | Sternum fracture.mp. | 24 |
| 3 | Blunt chest injury.mp. | 132 |
| 4 | Or/1-3 | 16385 |
| 5 | Exp mortality/ | 275077 |
| 6 | Exp Pneumonia/ | 69019 |
| 7 | “Outcome and Process Assessment (Health Care)”/ or “Outcome Assessment (Health Care)”/ | 56981 |
| 8 | “Length of stay”/ | 72647 |
| 9 | Or/ 5-8 | 453706 |
| 10 | 4 and 9 | 1128 |
| 11 | Limit 10 to (humans and yr=”1990 –Current” and “all adult (19 plus years)”) | 544 |
| CINAHL Database | | |
| 1 | (MH “Thoracic Injuries+”) | 1493 |
| 2 | (MH "Hospital Mortality") OR (MH "Cause of Death") | 10461 |
| 3 | (MH "Treatment Outcomes") OR (MH "Outcomes (Health Care)") | 133943 |
| 4 | 2 or 3 | 142697 |
| 5 | 1 and 4 | 147 |
| 6 | Limit 5 to all adult | 107 |
| EMBASE Database | | |
| 1 | Rib fracture/ | 4936 |
| 2 | Thorax blunt trauma/ | 2322 |
| 3 | Hospital acquired pneumonia/ or pneumonia/ | 117822 |
| 4 | Outcome assessment/ | 234313 |
| 5 | Mortality/ | 563924 |
| 6 | “length of stay”/ | 84835 |
| 7 | 1 or 2 | 6989 |
| 8 | Or/3-6 | 934349 |
| 9 | 7 and 8 | 907 |
| 10 | Limit 9 to (human and yr=”1990 –Current” and (adult <18 to 64 years> or aged <65+ years>)) | 430 |

**Table S3: Summary of literature searched**

| Date Searched | Database | Titles and Abstracts Reviewed | Full Article reviewed |
| --- | --- | --- | --- |
| March 2014 | Cochrane | 26 | 0 |
| March 2014 | Medline | 544 | 39 |
| March 2014 | EMBASE | 430 | 21 |
| March 2014 | CINAHL | 107 | 5 |
