# Supplementary material for: Treatments for blunt chest trauma and their impact on patient outcomes and health service delivery
Source: Scand J Trauma Resusc Emerg Med. 2015 Feb 8;23:17. doi: 10.1186/s13049-015-0091-5 (PMC4322452; doi:10.1186/s13049-015-0091-5)
Supplement: Additional file 2: Table S4. — Overview of the Studies. [file 13049_2015_91_MOESM2_ESM.docx]

**Additional File 2**

**Table S4: Overview of the Studies**

| **Author, date and country;** | **Type of Study** | | | | **Patient group** | **Outcomes** | | | **Main Findings** | **Level of Evidence*** | | | | | **Comments** |  |
| --- | --- | --- | --- | --- | --- | --- | --- | --- | --- | --- | --- | --- | --- | --- | --- | --- |
| Surgical Fixation | | | | | | | | | | | | | | | |  |
| Doben et al., 2014, USA^20^ | Retrospective cohort study | | | | 21 patients (rib fracture stabilization v controls) | ICU- LOS; LOS; total ventilator days | | | Decreased number of days of mechanically ventilation | Poor | | | Rib fixation was rescue technique not primary surgery | | |  |
| Nirula et al., 2006, USA^25^ | Retrospective case control | | | | 60 patients (surgical rib fixation v analgesia) | Ventilator days | | | Decreased mean number of ventilator days | Fair | | | Significantly higher incidence of head injury in control group | | |  |
| Althausen et al., 2011, USA^23^ | Retrospective case control | | | | 50 patients (surgical fixation v nonoperative management) | Time in ICU; ventilator requirement; hospital LOS; tracheostomy; pneumonia; reintubation; home O_2_ | | | Decrease in ICU LOS; decreased ventilator requirements; shorter hospital LOS; fewer tracheostomies; less pneumonia; less need for reintubation; Decreased home oxygen requirements | Fair | | |  | | |  |
| Marasco et al., 2013, Australia^28^ | Prospective, randomised controlled trial | | | | 46 patients (surgical fixation v nonoperative management) | Duration of mechanical ventilation; ICU stay | | | Reduction in ICU LOS; reduction in total ICU stay; reduction in duration of noninvasive ventilation post-extubation | Good | | | Randomisation did not account for smoking as a confounder; patient outcomes e.g. pain were not assessed | | |  |
| Voggenreiter et al., 1998, Germany^24^ | Retrospective, case-control study | | | | 42 patients (operative v nonoperative; pulmonary contusion v no pulmonary contusion) | Duration of ventilatory support; complications | | | Ventilatory time in surgical patients without pulmonary contusion was significantly shorter than surgical patients with pulmonary contusion and non-surgical patients without pulmonary contusion | Fair | | |  | | |  |
| De Moya et al., 2011, USA^58^ | Retrospective bi-institutional matched case-control study | | | | 48 patients (surgical fixation v control) | Amount of narcotics administered | | | Morphine requirement decreased preoperatively compared to postoperatively; no difference in mean morphine requirements | Fair | | | No standardisation of doses of NSAIDs and epidurals; no long term follow up | | |  |
| Tanaka et al., 2002, Japan^26^ | Prospective, randomised Study | | | | 37 patients (surgical fixation v internal pneumatic stabilization) | Ventilatory period; ICU stay; pneumonia | | | Shorter ventilatory period; shorter ICU LOS; lower incidence of pneumonia; increased forced vital capacity at 1 month; increased percentage of patients that returned to employment at 6 months | Good | | |  | | |  |
| Ahmed & Mohyuddin, 1995, United Arab Emirates^83^ | Retrospective, case-control study | | | | 64 patients (surgical fixation v mechanical ventilation) | Duration of assisted ventilation; pulmonary complications | | | Lower days of assisted ventilation; lower pulmonary complications - infection, septicaemia and barotraumas | Fair | | | No p values of significance | | |  |
| Granetzny et al., 2005, Egypt^27^ | Prospective, randomised, comparative study | | | | 40 patients (surgical fixation v chest wall binding) | Chest wall deformity; pulmonary functions two months post-operatively; pulmonary Complications | | | Decreased duration of mechanical ventilation days; decreased ICU and hospital stay; decreased pulmonary complications; improved pulmonary function tests | Good | | | Randomisation occurred within blocks of 10 patients; conservative group had higher associated injuries | | |  |
| Analgesia | | | | | | | | | | | | | | | |  |
| Hakim, Latif & Anis, 2012, Egypt^84^ | Randomised, parallel-arm, open label trial | 55 patients (lumbar v thoracic epidural) | | | | Mechanical ventilation; pneumonia | | | No difference | | Good | | No control for pain or respiratory compromise | | |  |
| Topcu, Ekici & Sakarya, 2007, Turkey^85^ | Retrospective case series | 49 patients (thoracic epidural v PCA) | | | | Pain scores; ICU LOS; mechanical ventilation requirements; pulmonary and cardiac complications. | | | Decreased ICU LOS; decreased pain score from the 6^th^ hour of therapy | | Fair | | Greater number of rib fractures in the thoracic epidural analgesia group | | |  |
| Truitt et al., 2011, USA^29^ | Prospective, case series | 177 patients (intercostal nerve block v control) | | | | Numeric Pain Score (NPS); respiratory rate; LOS | | | Decreased LOS; improvement in NPS at rest and coughing; decreased respiratory rate | | Fair | | Single institution, analgesia was not compared to another therapy | | |  |
| Bayouth et al., 2013, USA^8^ | Retrospective chart review | 42 patients (IV ibuprofen and narcotics v narcotics) | | | | Patient reported pain scores; mean pain requirements | | | Decreased mean daily morphine equivalent over first 7 days of hospitalisation; decreased total weekly morphine equivalent requirement; lower mean highest pain score; lower mean lowest pain score | | Fair | | No definition of how pulmonary complications were determined including pneumonia | | |  |
| Bulger et al., 2004, USA^36^ | Prospective, randomised trial | 46 patients (systemic opioid v epidural) | | | | Pneumonia; duration of mechanical ventilation; hospital and ICU LOS; mortality | | | 6.0 fold increase in the risk of pneumonia; 2.0 fold increase in ventilator days | | Good | | Cross-over allowed between the two groups | | |  |
| Gage et al., 2014, USA^11^ | Retrospective cohort study | 836 patients (epidural catheters v no epidural) | | | | Mortality; pulmonary complications | | | Patients with 3 or more rib fractures had lower mortality at 30, 90 and 365 days; the adjusted odds of death were 0.08, 0.09 and 0.12 respectively; no difference in pulmonary complications; increased ICU LOS | | Good | | Large difference in numbers between two cohorts | | |  |
| Mohta et al., 2009, India^12^ | Prospective, randomised comparison | 30 patients (epidural catheter v paravertebral block) | | | | Visual analogue scale (VAS) pain score; pulmonary complications | | | No difference in VAS; no difference in pulmonary complications; increased hypotension in epidural catheter | | Fair | |  | | |  |
| Wisner, 1990, USA^35^ | Retrospective chart review | 307 patients (IV narcotics v epidural) | | | | Mortality; pulmonary complications | | | Higher mortality rate in IV/IM narcotic group; odds of death were 38% less for patients with epidural pain relief than IV/IM | | Fair | | Large difference in sample size between groups | | |  |
| Asha, Curtis, Taylor & Kwok, 2013, Australia^31^ | Retrospective, cohort study | 227 patients (PCA v interval dosing) | | | | Complications; hospital LOS; cost | | | No significant findings | | Fair | | Large difference in sample size between groups | | |  |
| Shukla et al., 2008, Malaysia^40^ | Retrospective, non-randomised case series | 11 patients (paravertebral block v initial bolus) | | | | Pain score at rest, on vital capacity and cough compared | | | Improved pain scores after 30 mins at rest, on vital capacity maneuver and on cough | | Poor | | Not comparable to other regional analgesia techniques | | |  |
| Ingalls et al., 2010, USA^33^ | Randomised, double-blind, placebo controlled trial | 58 patients (lidocaine patch 5% v placebo) | | | | Total (IV and oral) narcotic used; non narcotic pain medication; average pain score; pulmonary complications; LOS | | | No significant findings | | Good | | Low dosing of the lidocaine patch | | |  |
| Yeh, Kutcher, Knudson & Tang, 2012, USA^3^ | Retrospective review | 187 Patients (thoracic epidural v PCA) | | | | Pulmonary complications; ICU and Hospital LOS | | | Longer ICU and hospital LOS; more total ribs fractured; higher incidence of bilateral rib fractures | | Fair | | Higher severity of rib fractures in epidural group | | |  |
| Wu, Jani, Perkins & Barquist, 1999, USA^37^ | Retrospective study | 64 patients (epidural analgesia v PCA) | | | | Pain scores; complications: need for intubation, pneumonia, cardiac complications; LOS | | | Lower pain ratings at all time intervals with the exception of baseline (0 hour) scores; No difference in LOS | | Fair | | Epidural group had more rib fractures | | |  |
| Kieninger et al., 2005, USA^86^ | Retrospective chart review | 187 Patients (epidural v IV narcotics) | | | | Hospital LOS; Pulmonary complications | | | Epidural group (with low ISS) had a longer LOS; Higher complications in epidural group (with high ISS) | | Fair | |  | | |  |
| Karmakar et al., 2003, Hong Kong^15^ | Prospective, non-randomised case series | 15 patients (paravertebral block) | | | | Pain Score; pulmonary Function Test | | | Improvements in pain scores after the initial treatment compared to before treatment | | Poor | | No control group | | |  |
| Zink et al., 2011, USA^39^ | Retrospective case control study | 58 patients (lidocaine v control) | | | | Pain scores; narcotic use. | | | Lower pain score at 24 hours after placement; lower pain score at 60 days; no difference in narcotic use | | Fair | | Those with more fractures were more likely to receive patches | | |  |
| Pierre et al., 2005, USA^87^ | Unknown | 22 patients: (epidural v PCA) | | | | Hospital LOS; pain score | | | Decreased hospital and ICU LOS; lower pain scores at 24 and 36 hours | | Poor | | Abstract only | | |  |
| Mackersie et al., 1991, USA^30^ | Prospective randomised trial | 32 patients (epidural v IV) | | | | Ventilatory function tests; arterial blood gases; visual analog scores | | | Epidural group: improved maximum inspiratory pressure and vital capacity  IV fentanyl: improved vital capacity; changed ABGs - Increase in PaCO_2_ and decrease in PaO_2_ | | Good | |  | | |  |
| Moon et al., 1999, USA^34^ | Prospective, randomised trial | 24 patients (epidural v PCA) | | | | Catecholamine plasma levels; Verbal pain score; maximum inspiratory force and tidal volume | | | Reduced plasma levels of IL-8 on days 2 and 3; reduced verbal rating score of pain on days 1 and 3; improved maximal inspiratory force and tidal volume on day 3 | | Fair | | Of 34 patients originally enrolled, 10 dropped out. | | |  |
| Clinical Pathway | | | | | | | | | | | | | | | |  |
| Todd et al., 2006, USA^45^ | Prospective, observational cohort study | 300 patients (clinical pathway v control) | | | | Pain management; ICU and hospital LOS; pneumonia; mortality | | | More PCA prescribed; increased epidural catheter utilisation; decreased shock trauma ICU LOS; decreased hospital LOS; decreased pneumonia; decreased mortality | | Good | | Study sample collected over 5 years – changes in practice guidelines during this time not just implementation of clinical pathway | | |  |
| Adrales et al., 2002, USA^46^ | Cohort Study | 61 patients (pre and post practice guidelines) | | | | Duration of Thoracostomy tube; pulmonary complications | | | Post-practice guidelines group: 3 fewer days of thoracostomy tube; less Chest radiographs | | Fair | | Limited sample size of pre-practice guidelines group | | |  |
| Sesperez et al., 2001, Australia^41^ | Prospective consecutive study | 235 patients | | | | Variances to elements of care | | | Increase in the outcomes achieved between before and after implementation; the elements that incurred the greatest number of variances were assessment, elimination, pain management, and medications | | Good | | Positive variances were not recorded | | |  |
| Wilson et al., 2001, Australia^42^ | Prospective consecutive study | 146 patients | | | | Applicability to pathway | | | Applicability of fractured ribs to a clinical pathway – 93.4% | | Good | | Limited discussion on rib fracture pathway procedure | | |  |
| Menditto et al., 2012, Italy^43^ | Retrospective, before-after study | 240 patients (pre- pre-Emergency Department observation unit (EDOU) period v post) | | | | Mortality or complication; admission to hospital; LOS; ED readmission; cost | | | In Pre-EDOU period: increased LOS; increased rate of hospitalisation 49% v 24%; increased ED readmittance  Post-EDOU period: increased thoracostomy performed in admitted patients | | Good | | Co-morbidities were not evaluated; study in third level hospital | | |  |
| Sahr et al., 2013, USA^44^ | Retrospective, before-after study | 148 patients (pre v post protocol) | | | | Hospital and ICU LOS | | | Decrease in hospital LOS from pre-protocol to post-protocol intervention for patients with 3 or more fractured ribs | | Good | | Small group of patients at a single institution | | |  |
| Ventilation | | | | | | | | | | | | | | | |  |
| Walz, Mollenhoff & Muhr, 1998, Germany^48^ | Prospective study | | 30 patients (CPAP v no CPAP) | | | | Pneumonia; ICU stay | | No difference in pneumonia; decreased ICU LOS | | Poor | | Abstract only in English | | |  |
| Bolliger & van Eeden, 1990, South Africa^47^ | Randomised, controlled trial | | 69 patients (Mechanical ventilation v CPAP) | | | | ICU and hospital LOS; pulmonary complications | | Longer ICU LOS; longer hospital LOS; increased complications 73% v 28% | | Fair | | Groups not matched for ISS | | | |
| VATS | | | | | | | | | | | | | | | | |
| Fabbrucci et al., 2008, Italy^50^ | Retrospective, consecutive study | | 81 patients (chest tube v chest tube and VATS) | | | | Postoperative complications; indwelling thoracic drainage time; LOS; blood transfusion; mortality | | Lower blood transfusion 4 patients with 2-4 units v 15 patient with 2-20units; shorter drainage time 5.7days v 6.3days | | Poor | | Descriptive results; Higher ISS in chest tube alone group | | | |
| Smith et al., 2011, USA^51^ | Retrospective review | | 83 patients (VATS ≤ 5 days after injury v VATS > 5 days after injury) | | | | Hospital LOS; conversion to thoracotomy | | Lower rate of conversion to an open procedure; lower overall hospital LOS | | Fair | |  | | | |
| Rib Belt | | | | | | | | | | | | | | | | |
| Quick, 1990, USA ^52^ | Prospective, randomised study | | | 20 patients (analgesia v rib belt and analgesia) | | | Pulmonary function; pulmonary complications | | No difference between groups | | | Poor | |  | | |
| Multimodal therapy; Spirometry | | | | | | | | | | | | | | | | |
| Schwed et al., 2013, USA^59^ | Retrospective review | | | | 107 patients (multimodal v individual therapy) | | | Complications | Multimodal therapy was not associated with fewer complications; incentive spirometry reduced morbidity | | | Fair | | Abstract only published | | |

Legend: * Level of evidence: Determined using the US preventive services task force recommendations for quality of evidence^18^. This scoring system was chosen as it appraises evidence from a variety of research designs^88^.

ICU: Intensive care unit; LOS: Length of stay; O_2_: Oxygen gas; PCA: Patient controlled analgesia; NPS: Numeric pain score; IV: Intravenous; VAS: Visual analogue scale; IM: intramuscular; ISS: Injury severity score; ABG: Arterial Blood gas; PaCO_2_: Partial pressure of carbon dioxide; PaO_2_: Partial pressure of oxygen; EDOU: Emergency department observation unit; ED: Emergency department; CPAP: Continuous positive airway pressure; VATS: Video-assisted thoracoscopy surgery
