# Supplementary material for: Treatments for blunt chest trauma and their impact on patient outcomes and health service delivery
Source: Scand J Trauma Resusc Emerg Med. 2015 Feb 8;23:17. doi: 10.1186/s13049-015-0091-5 (PMC4322452; doi:10.1186/s13049-015-0091-5)
Supplement: Additional file 3: Table S5. — Summary of each treatment option and strength of the literature [83-88]. [file 13049_2015_91_MOESM3_ESM.docx]

**Additional File 3**

**Table S5: Summary of each treatment option and strength of the literature**

| **Treatment Option** | **Comment** |
| --- | --- |
| Surgical Fixation | Across the literature there were consistent improvements in patients with flail chest and surgical fixation with fewer days of mechanical ventilation, ICU-LOS and cost savings compared to non-operative techniques. Three out of nine studies were randomized controlled trials, and the level of evidence in all studies was primarily fair or good. |
| Patient Controlled Analgesia | PCA compared to epidural analgesia is less effective for pain control. The evidence is limited to primarily retrospective studies with relatively small sample sizes. |
| Epidural Analgesia | Epidural analgesia reduced complications and mortality compared to other analgesic techniques. Epidural analgesia compared to intravenous narcotics had a good level of evidence, and included three randomised controlled trials. |
| Lidocaine Patches | Lidocaine patches were not effective at improving pain control or hospital length of stay, which was supported by a good level of evidence. |
| Nerve Blocks | Nerve blocks improve patient pain scores, however, in studies that compared nerve blocks to a control, the level of evidence was poor. |
| Clinical Pathways | Clinical pathways improve patient and health service outcomes, by reducing patient complications and hospital costs, as it encouraged a multidisciplinary response and early and aggressive analgesia. The level of evidence was overall good, however, no randomized controlled trials can be conducted. |
| Ventilation | Non-invasive ventilation such as CPAP reduces complications and hospital LOS compared to mechanical ventilation. However, mechanical ventilation is the mainstay treatment for patients with concomitant injuries. No studies in this comparison had a good level of evidence. |
| Video-assisted Thoracoscopy Surgery | VATS is effective for treating the complications of blunt thoracic injury however is not an immediate therapy. The level of evidence was fair or poor and both studies were retrospective reviews. |

ICU: Intensive care unit; LOS: Length of stay; PCA: Patient controlled analgesia; CPAP: Continuous positive airway pressure; VATS: Video-assisted thoracoscopy surgery
